# Exploring the Therapeutic Potential of *Petiveria alliacea* L. Phytochemicals: A Computational Study on Inhibiting SARS-CoV-2’s Main Protease (Mpro)

**DOI:** 10.3390/molecules29112524

**Published:** 2024-05-27

**Authors:** Md. Ahad Ali, Humaira Sheikh, Muhammad Yaseen, Md Omar Faruqe, Ihsan Ullah, Neeraj Kumar, Mashooq Ahmad Bhat, Md. Nurul Haque Mollah

**Affiliations:** 1 Bioinformatics Laboratory, Department of Statistics, Faculty of Science, University of Rajshahi, Rajshahi 6205, Bangladesh; ahad.chembd@gmail.com; 2 Department of Chemistry, Faculty of Science, University of Rajshahi, Rajshahi 6205, Bangladesh; 3 Department of Chemistry, Faculty of Science, Bangabandhu Sheikh Mujibur Rahman Science & Technology University, Gopalganj 8100, Bangladesh; humairasheikh.bd@gmail.com; 4 Institute of Chemical Sciences, University of Swat, Main Campus, Charbagh 19130, Pakistan; ihsanmtk@uswat.edu.pk; 5 Department of Computer Science and Engineering, Faculty of Engineering, University of Rajshahi, Rajshahi 6205, Bangladesh; faruqe.cse@gmail.com; 6 Department of Pharmaceutical Chemistry, Bhupal Nobles’ College of Pharmacy, Udaipur 313001, Rajasthan, India; neerajkumarkamra@gmail.com; 7 Department of Pharmaceutical Chemistry, College of Pharmacy, King Saud University, Riyadh 11451, Saudi Arabia; mabhat@ksu.edu.sa

**Keywords:** SARS-CoV-2 infections, main protease, phytocompound, toxicity, computational approaches

## Abstract

The outbreak of SARS-CoV-2, also known as the COVID-19 pandemic, is still a critical risk factor for both human life and the global economy. Although, several promising therapies have been introduced in the literature to inhibit SARS-CoV-2, most of them are synthetic drugs that may have some adverse effects on the human body. Therefore, the main objective of this study was to carry out an in-silico investigation into the medicinal properties of *Petiveria alliacea* L. (*P. alliacea* L.)-mediated phytocompounds for the treatment of SARS-CoV-2 infections since phytochemicals have fewer adverse effects compared to synthetic drugs. To explore potential phytocompounds from *P. alliacea* L. as candidate drug molecules, we selected the infection-causing main protease (Mpro) of SARS-CoV-2 as the receptor protein. The molecular docking analysis of these receptor proteins with the different phytocompounds of *P. alliacea* L. was performed using AutoDock Vina. Then, we selected the three top-ranked phytocompounds (myricitrin, engeletin, and astilbin) as the candidate drug molecules based on their highest binding affinity scores of −8.9, −8.7 and −8.3 (Kcal/mol), respectively. Then, a 100 ns molecular dynamics (MD) simulation study was performed for their complexes with Mpro using YASARA software, computed RMSD, RMSF, PCA, DCCM, MM/PBSA, and free energy landscape (FEL), and found their almost stable binding performance. In addition, biological activity, ADME/T, DFT, and drug-likeness analyses exhibited the suitable pharmacokinetics properties of the selected phytocompounds. Therefore, the results of this study might be a useful resource for formulating a safe treatment plan for SARS-CoV-2 infections after experimental validation in wet-lab and clinical trials.

## 1. Introduction

The novel coronavirus ‘SARS-CoV-2’ is the main cause of the COVID-19 pandemic that has spread around the world from the city of Wuhan in China since late 2019. It is still a major threat to public health and the global economy. This virus is a positive-sense RNA virus that does not have any segments and is enveloped. The main four structural proteins in SARS-CoV-2 are the nucleocapsid protein (N), the membrane glycol protein (M), the small envelope glycoprotein (E), and the spike protein (S) [1]. The S-protein forms homotrimers that stick out from the virus’s surface and interact with the angiotensin-converting enzyme-2 (ACE2) receptor in host cells. The N-protein is responsible for encapsulating the viral genome RNA into a long ribonucleocapsid (RNP) complex and assisting in the assembly of the virion through interaction with the viral genome and M-protein. The membrane shape of this virus is controlled by the M-protein, which is responsible for the stability of the N protein–RNA complex. The last structural E-protein is essential for viral maturation and generation [2]. Once fusion and entry are accomplished successfully, the viral genome is released. From the genomic RNA, ORF1a and ORF1b are translated into two types of bulky polyproteins, pp1a and pp1ab, necessary for viral replication and transcription, which are subsequently cleaved into multiple functional viral proteins by viral proteases, specifically the main protease (Mpro), also known as 3-chymotrypsin-like protease (3CLpro), that encodes nsp3 and nsp5. Mpro is a protease widely recognized for its crucial role in the enzymatic activity that leads to replicate protein post-translational processing [3]. Therefore, Mpro/3CLpro is considered a promising therapeutic target in order to explore drug molecules for the treatment of SARS-CoV-2 infections [4,5,6]. 

As of 22 April 2024, the SARS-CoV-2 virus has spread to 230 countries worldwide and infected around 704,753,890 patients, of whom 675,619,811 (99%) are reported to have recovered, 7,010,681 (1%) have died, and the remaining 22,123,398 are actively infected. Of the active cases, 22,088,604 (99.8%) are in mild condition and the remaining 34,794 (0.2%) are in severe or critical condition [7]. Evidently, COVID-19 is still out of control due to random mutations in the SARS-CoV-2 genome, although most people around the world are already vaccinated by WHO-approved vaccines (Moderna, Sinopharm, Pfizer, Sinovac-CoronaVac, COVISHEILD, and Janssen). As remedies, potential candidate drugs are also required to reduce the death rate associated with COVID-19. Currently, some FDA-approved synthetic drugs are available and are described in the literature for the treatment of SARS-CoV-2 infections, including ritonavir and lopinavir [8], minocycline [9], oseltamivir [10], corticosteroids [11], tocilizumab [12], ribavirin and riboflavin [13], ciclesonide [14], and niclosamide [15]. However, synthetic drugs might have some adverse effects on the human body [16,17]. In this context, phytochemical/phytocompound-based natural medicine may have comparatively fewer adverse effects on the human body than synthetic drugs [16]. Therefore, it is necessary to explore different alternative phytochemicals/phytocompounds as a natural medicine for the treatment of SARS-CoV-2 infections. *Petiveria alliacea* L. is a well-known medicinal plant, and some of its phytochemicals, including benzaldehyde, dibenzyl sulfide, coumarin, isoarborinol, alpha-pinene, beta-pinene, nerolidol, allantoin, and daucosterol, are considered antiviral, anti-inflammatory, analgesic, anti-HIV, antidiabetic, antitumor, anti-cancer, and antimicrobial drug molecules [18,19,20,21,22,23,24,25]. However, so far, there is no study in the literature that has explored the phytochemicals of *P. alliacea* L. as drug molecules for the treatment of SARS-CoV-2 infections. Therefore, this study aimed to investigate the anti-SARS-CoV-2 activity of *P. alliacea* L. phytocompounds through bioinformatics analysis. 

## 2. Materials and Methods

**Figure 1:**
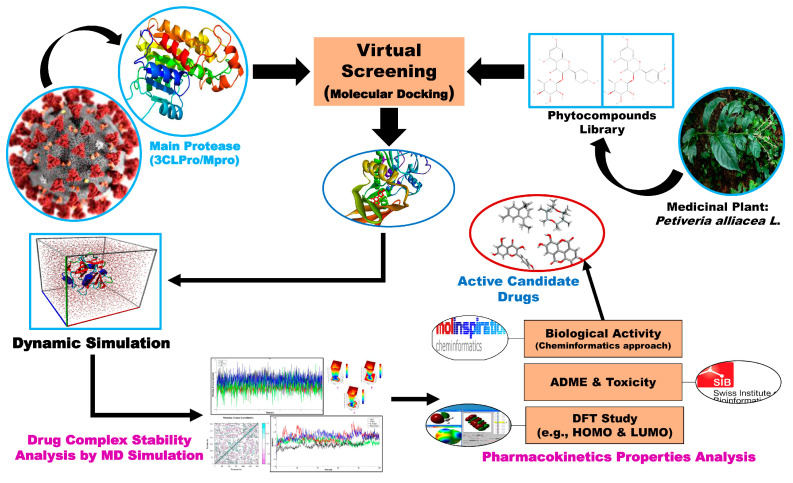
Graphical representation of the current research.

In the case of drug discovery, the identification of drug targets and the identification agents/ligands (small molecules) are equally important. This study considered the SARS-CoV-2 infection-causing main protease (Mpro) as the drug target and the phytochemicals of *Petiveria alliacea* L. to explore potential drug molecules for the treatment of SARS-CoV-2 infections by using integrated bioinformatics approaches. The workflow of this study is given in Figure 1.

### 2.1. Target Protein Retrieval and Preparation

**Figure 2:**
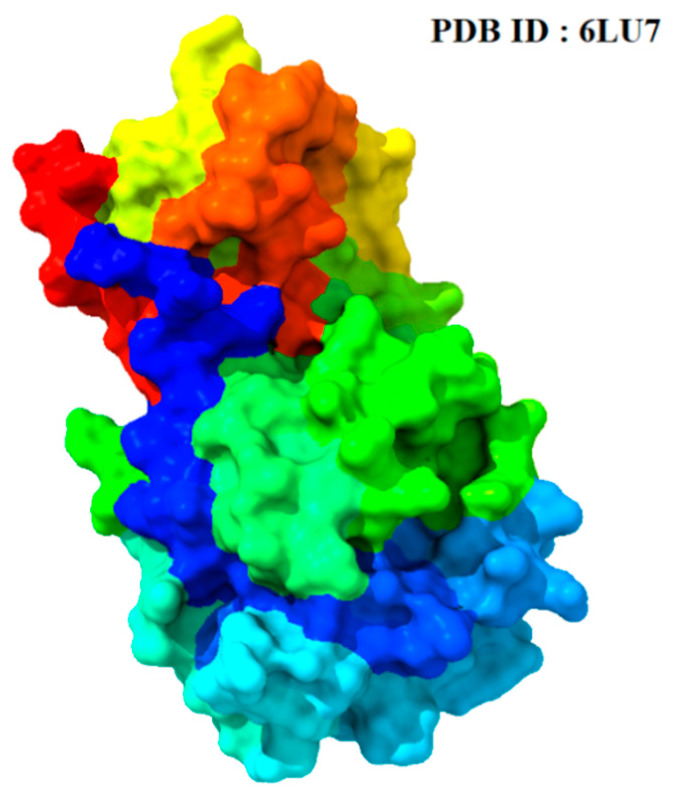
The 3D conformational view of the target protein Mpro (PDB ID: 6LU7) from SARS-CoV-2 virus, colored by amino acid type. Such as, Red: Hydrophobic, Green: Polar, Blue: Positively charged, Magenta: Negatively charged.

The X-ray crystallography structure of the main protease (Mpro/3CL pro) was retrieved from the RCSB protein database with its PDB ID 6LU7 [26]. The missing residues and the sequence of the retrieved protein were observed by using PyMOL (Academic) v2.5.5 [27]. The 3D crystal structure of Mpro was prepared by removing existing heteroatoms, ligands, and water molecules in BIOVIA Discovery Studio software 2021 Client [28]. Finally, the protein structures were minimized by using swiss PDB viewer (spdbv) software v4.1.0 [29]. The graphical representation of the 3D structure of Mpro (6LU7) is given below (Figure 2).

### 2.2. Phytocompound Collection and Preparation

In this study, we collected 150 phytocompounds for *Petiveria alliacea* L. through a systematic literature review [24,30,31,32,33] and using the online database IMPPAT 2.0 [34] (https://cb.imsc.res.in/imppat/home, accessed on 10 January 2024). The 3D structure of these phytocompounds was extracted from the PubChem database [35]. After that, the energy of the ligands was optimized by using a virtual screening tool, PyRx 0.8*v*, with a universal force field (UFF) at 500 steps [36]. For further study, we converted all of the phytochemicals into pdbqt from sdf format through the open babel tool plugin in PyRx [37,38].

### 2.3. Molecular Docking

Molecular docking is an effective approach for exploring receptor protein-guided potential drug molecules for the treatment of disease. The Auto-Dock Vina [39] plugin PyRx tool was used to perform molecular docking of the target protein with the phytocompounds (ligands) of *Petiveria alliacea* L. Then, the phytocompounds were ordered according to their binding affinity scores. The molecular interactions for the top-ranked protein–ligand complexes were visualized using BIOVIA Discovery Studio 2021 [28]. 

### 2.4. Molecular Dynamic Simulation

Molecular dynamics (MD) simulations with YASARA software [40] (v16.9.) were performed to investigate the stability of the top-ranked protein–ligand complexes. Each of these complexes was investigated for 100 ns MD simulation under precise circumstances, including a pH of 7.4, a 298 K temperature, and a solvent density of 0.997 [41]. The AMBER14 force field [42] was used with the default simulation settings. During the simulation, the ligand within the complex was subjected to an acceleration control force of 1000 pm/ps2 (picometer/picosecond square) [43]. The evaluation was performed by evaluating the backbone alpha carbon or side chain atom. We investigated the root mean square deviation (RMSD), root mean square fluctuation (RMSF), and dynamic cross-correlation matrix (DCCM) of protein–ligand complexes to better understand structural deviation and fluctuation in dynamic situations. This study sheds light on how a ligand binding to a protein’s active site influences its capacity to reach equilibrium [44]. The RMSD and RMSF figures of each complex were plotted according to the following equations [45]:

(1) $$\mathrm{RMSD}=\sqrt{\frac{\sum_{i=1}^{N}R_{i}\times R_{i}}{n}}$$

where RMSD = root mean square deviation; *i* = variables; and *R_i_* = the vector linking the positions of atom *i* (of *N* atoms) in the reference snapshot and the current snapshot after optimal superposition, and

(2) $$\mathrm{RMSF}=\sqrt{\sum_{j=1}^{3}\left(\frac{1}{N}\;\sum_{k=1}^{N}P_{ikj}^{2}-{\bar{P}}_{ikj}^{2}\right)}$$

where RMSF = root mean square fluctuation; the RMSF of atom *i* with *j* running from 1 to 3 for the *x*, *y*, and z coordinate of the atom position vector *P* and *k* running over the set of *N* evaluated snapshots. 

#### MM-PBSA-Based Binding Energy Analysis

The molecular mechanics Poisson–Boltzmann surface area (MM-PBSA) technique was used to calculate the binding free energy (BFE) of the selected protein–ligand complexes. This was accomplished by using trajectory snapshots from the MD simulation results by using YASARA [40]. The given equation was used to evaluate the BFE:

Binding free energy = E_potRecept_ + E_solvRecept_ + E_potLigand_ + E_solvLigand_ − E_potComplex_ − E_solvCom_

In drug design, it is used to assess the strength of the interaction between a drug candidate and its target protein. Negative binding energies indicate favorable binding or stable interaction, while positive binding energies indicate unstable interaction or unfavorable binding.

### 2.5. Principal Component Analysis (PCA)

Principal component analysis (PCA) was used to identify the molecular motion modes that predominate in the dynamic simulation’s trajectory. The generation of a covariance matrix classifies the atomic motions. The simulation trajectories were conducted using GROMACAS [46] with the help of a previously reported method [47,48,49]. The computations for each simulation trajectory were performed using the R environment [50] and the Bio3d package 2.3.0 for MD trajectory analysis [51]. 

#### Gibbs Free Energy Landscape (FEL) Analyses

The results from the PCA analysis were further analyzed using Gibbs free energy landscape (FEL) analyses. Free energy landscape (FEL) is used for 3D conformational sampling. The FEL was created in PyMOL using the geo-measure tool [52]. It uses the *gmx_sham* command to make 3FEL energy to calculate the joint probability distribution in the three-dimensional space.

### 2.6. ADMET and Druglikeness Properties Analysis

In-silico validation of the selected top-ranked phytocompounds was considered based on their ADMET (absorption, distribution, metabolism, excretion, and toxicity) and drug-likeness properties analysis. Lipinski’s rule, a widely used method, was used to examine the drug-likeness properties [53]. The ADMET profile was evaluated by using the web tool ADMETlab 2.0 [54]. The toxicity evaluation was conducted using the online web application pkCSM [55]. The evaluation of toxicity is critical in drug development since it directs the determination of the harmful dosage in animal model studies, reducing the number of such investigations. This study also examined model factors such as lipophilicity (LogP), rate of oral absorption, Caco2, CNS, logPo/w, MDCK, logHERG, LogBB, and percentage of human oral absorption from pharmacokinetic research [41].

### 2.7. Prediction of Biological Activity

To investigate the biological activities of the top-ranked phytocompounds, a cheminformatics web tool available online, “Molinspiration” (https://www.molinspiration.com/, accessed on 5 February 2024), was used [56]. The SMILE of the selected phytocompounds was retrieved from PubChem and used as input files to evaluate biological activity. Based on the study’s findings, a fragment-based model was established to calculate the bioactivity of each substructure fragment and the molecule fragments’ total activity.

### 2.8. Density Functional Theory Analysis

The three top-ranked phytocompounds from the molecular docking analysis were selected to evaluate their structural and functional properties and their quantum chemical properties by using density functional theory (DFT) analysis. It is widely used to calculate the energies of frontier molecular orbitals (FMO), electronic and structural properties, and thermodynamic energies in the gaseous state of the compounds by applying the Becke–3-parameter Lee–Yang–Parr (B3LYP) [57] method with the basis set 6–311 G [58] on Gaussian-09 software programs [43,59,60,61,62]. In this study, the energy of the highest occupied molecular orbital (HOMO), the energy of the lowest unoccupied molecular orbital (LUMO), the energy gap (∆E), electron affinity, electrophilicity index, hardness, softness, and other parameters were calculated for the three drug molecules associated with the top-scorer complexes by using Gaussian-09 and GaussView-6 parameters [63]. The following are the mathematical definitions of these parameters [64]:

∆E = E_LUMO_ − E_HOMO_ (3)

I = −E_HOMO_ (4)

A = −E_LUMO_ (5)

(6) $$\eta =\frac{I-A}{2}$$

(7) $$\sigma =\frac{1}{\eta }$$

(8) $$\chi =\frac{I+A}{2}$$

µ = − χ (9)

## 3. Results and Discussion

### 3.1. Molecular Docking between Mpro and Phytocompounds

In this study, 150 phytocompounds of *P. alliacea* L. that were found during the literature review were considered as drug agents/ligands for molecular docking with the main protease (Mpro) of SARS-CoV-2. Table S1 shows the 35 top-ranked receptor–ligand binding affinity scores. The results showed that myricitrin, friedelanol, engeletin, and astilbin produce higher binding affinity scores of −8.9, −8.9, −8.7, and −8.3 kcal/mol, respectively, with the receptor of Mpro, which indicates that they are top-ranked drug molecules. According to the ligand–protein interaction analysis, the docked poses clearly showed that phytocompounds bound to the cavity of the SARS-CoV-2 macromolecule structures.

#### 3.1.1. Molecular Interaction of Top-Ranked Protein–Ligand Complexes

**Figure 3:**
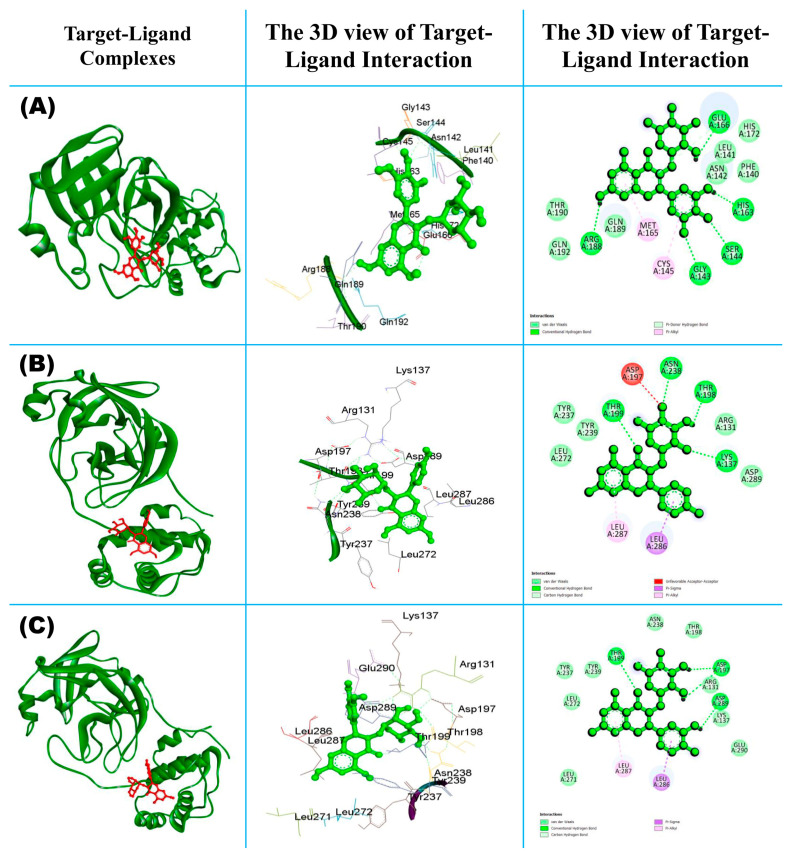
Display of post-docking analysis and ligand–protein interactions: (**A**) myricitrin vs. Mpro, (**B**) engeletin vs. Mpro, and (**C**) astilbin vs. Mpro.

Figure 3A exhibits the 3D and 2D views of the myricitrin–Mpro complex, highlighting the interacting amino acid residues of Mpro with the myricitrin ligand. This figure shows that both hydrophobic and hydrophilic bonds are involved in the interaction, including GLU:166, HIS:163, SER:144, GLY:143, and ARG:188, which interact through conventional hydrogen bond formation, whereas MET:165 and CYS:145 interact through Pi-Alkyl non-bonding interaction, and other residues are involved due to weak van der Waals force (Table S3). In the case in Figure 3B showing the complex of engeletin and Mpro, it can be clearly seen that the non-bonding interaction between the ligand and protein formed both favorable and unfavorable non-bonding interactions through both hydrophilic and hydrophobic bond formation. The interacting residues including ASN:238, THR: 198, LYS:137, and THR:199 are formed via conventional hydrogen bonding with the ligand molecule. Both LEU:287 and LEU:286 formed Pi-Alkyl and Pi-Sigma, respectively. The amino acid residue ASP:197 formed an unfavorable donor–donor non-bonding interaction with the selected drug molecule; this means that the interaction between the donor atoms (potentially hydrogen bond donors) of ASP:197 and the ligand molecule is not optimal. This unfavorable interaction might hinder the binding affinity between the protein and the ligand or affect the stability of the complex [65,66]. Figure 3C shows the ligand–protein interaction for the complex of astilbin and Mpro. From the 2D image, we can observe various types of interaction including hydrophobic, hydrophilic, and van der Waals force of attraction. The interacting amino residues ASP-197, ASP-289 and THR-199 are formed through conventional H-bond interaction (green color) with the target protein, which is known as a hydrophilic interaction; other non-bonding interactions are known as hydrophobic, such as Pi-Sigma (LEU-286) and Pi-Alkyl (LEU-287) bonding.

#### 3.1.2. Performance against Some Other SARS-CoV-2 Infection-Causing Genes

**Figure 4:**
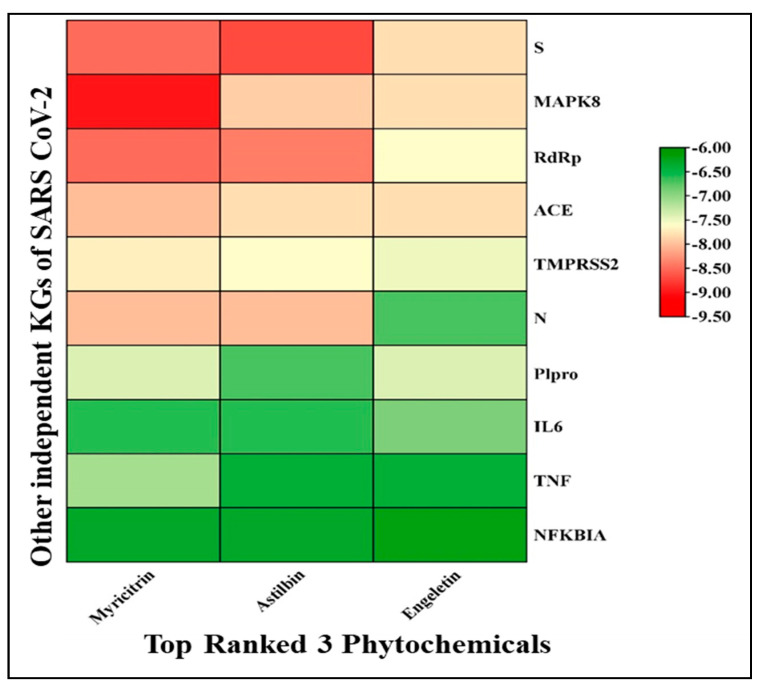
Molecular docking representation of the three top-ranked phytochemicals with other independent proteins of SARS-CoV-2.

To investigate the performance of the three proposed top-ranked phytocompounds (myricitrin, engeletin, and astilbin) in the inhibition of some other SARS-CoV-2 infection-causing genes, we selected the 10 top-ranked key genes/proteins (ACE2, Spike, NFKBIA, MAPK8, N, TNF, TMRSS2, RdRp, PLpro, and IL6) during the systematic literature review (Table S4). The docking study showed that at least one of these three proposed drug molecules significantly bind (score < −7.0 Kcal/mol) to these independent proteins except for NFKBA (Figure 4, Table S2). 

From Figure 4, it can be observed that myricitrin, astilbin, and engeletin form a good bond with the top-ranked proteins, showing strong binding with a high affinity score indicated with red cubic cells, in which NFKBIA binds with all of the ligands strongly compared to other proteins. Therefore, the three proposed top-ranked phytochemicals may inhibit some other SRAS-CoV-2 infection-causing genes as well.

### 3.2. Molecular Dynamics Simulation

From the molecular docking study, the three top-ranked protein–ligand complexes were considered for further molecular dynamics (MD) simulation analysis. MD simulation is a powerful method for studying the dynamic performance of molecular systems, and it is commonly used in drug design to anticipate drug–target interactions. To concisely examine the findings of an MD simulation, many key parameters (such as RMSF, RMSD, SASA, Rg, MM-PBSA, PCA, and FEL) that provide insight into the stability and behavior of the molecular system must be considered. 

#### 3.2.1. Root Mean Square Deviation (RMSD)

**Figure 5:**
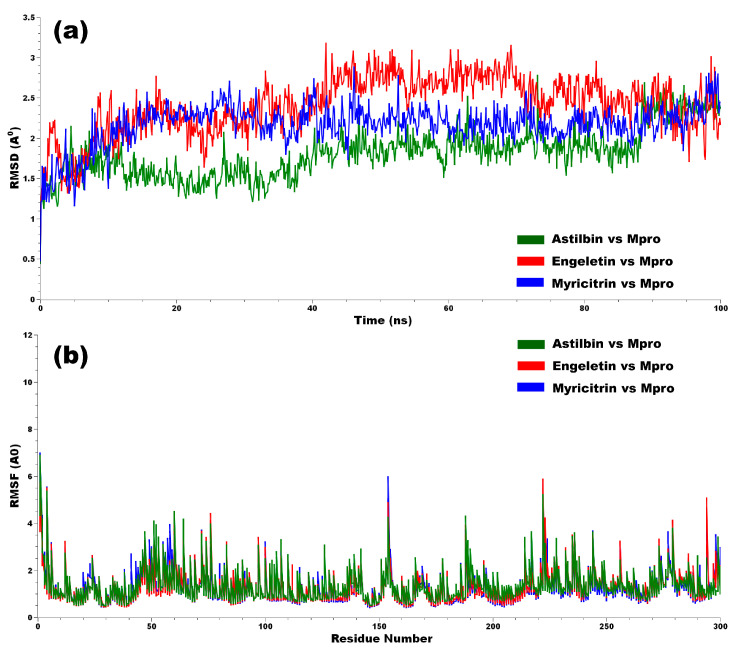
(**a**) Graphical presentation of the RMSD of the backbone atoms (C, Cα, and N) for each docked complex, and (**b**) the RMSF evaluated from the average RMSF of the atoms constituting the residue.

During the 100 ns MD simulation, the configurational changes of all protein–ligand complexes were evaluated in terms of root mean square deviation (RMSD). The RMSD plot for all of the protein–ligand complexes is shown in Figure 5a. RMSD is always non-negative, and a zero value implies perfect fit to the data, which is often achieved in practice. The RMSD value of the protein–ligand complexes was noted to understand the structural deviation in the dynamic condition. It also explains how a protein’s capability to approach equilibrium is influenced by the binding to its active site [67]. From Figure 5a, it was observed that the myricitrin vs. Mpro and astilbin vs. Mpro complexes seem to be stable within the range of 1.5–2.5 A^0^, whereas the approximate standard deviation range from the backbone is 2.0~2.5 A^0^ [68]. But, in the case of engeletin vs. Mpro, the deviation is higher than 2.0 A^0^, which is considered as moderately stable in its position; sometimes, it can be close to 3.0~3.3 A^0^ for the RMSD of a protein structure [69]. Thus, it can be assumed that the targeted ligands seem to be stable with the target protein’s structure.

#### 3.2.2. The Root Mean Square Fluctuation (RMSF)

In MD simulations, the RMSF is an important measure of the mobility and flexibility of protein–ligand complexes. Figure 5b illustrates the results of the study’s individual RMSF calculations for the protein–ligand complexes with the top three phytocompounds. A residue or atom with a lower RMSF value exhibits increased rigidity, while one with a higher RMSF value demonstrates enhanced flexibility or mobility. The secondary conformations of Mpro protease remain stable during the total run time of the MD simulation. The calculated average RMSF values for the astilbin–Mpro, engeletin–Mpro, and myricitrin–Mpro complexes were noted as 1.40, 1.32, and 1.30 angstrom (A^0^), respectively, (Table 1). From the RMSF results, it is clearly indicated that the complexes were stable, and hence, these phytocompounds may have the effect of reducing the catalytic action of SARS-CoV-2 infections.

#### 3.2.3. The Radius of Gyration (Rg)

**Figure 6:**
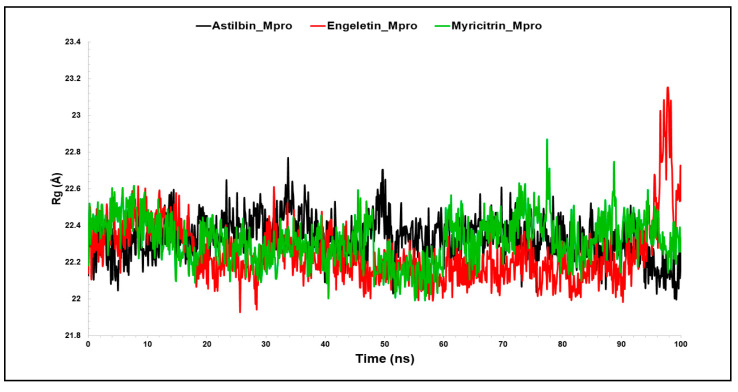
Representation of the Rg plot showing the changes observed in the conformational behavior of the all protein–ligand complex of aastilbin–Mpro, engeletin–Mpro, and myricitrin–Mpro.

Due to the presence or absence of ligands, the radius of gyration (Rg) indicates the degree of density of the protein structure [70]. Figure 6 illustrates a time-dependent graph of Rg for all of the protein–ligand complexes. Rg is usually used to figure out whether the complexes are folded or unfolded in a stable manner during the simulation time. The Rg may be used to identify the folding and unfolding of protein structure following the interaction of the ligands and proteins. In conclusion, a larger Rg indicates that the protein–ligand combination is less compact (more unfolded) [4,71]. The average Rg values of the astilbin–Mpro, engeletin–Mpro, and myricitrin–Mpro complexes were determined to be 22.34 nm, 22.24 nm, and 22.32 nm, respectively, which were significantly similar when compared with each other (Figure 6). As mentioned before, a protein can be considered to be stably folded if it is anticipated to retain a reasonably constant value of Rg during MD simulation. The Rg is regarded as unfolded if its value changes over time. Subsequently, all of the complexes exhibited nearly identical compactness and consistent levels for the Rg (Table 1), suggesting that the complexes are precisely overlaid and possess comparable compactness and stability qualities. It was observed that all of the compounds exhibited a very stable folded shape during the whole simulation period, under the typical conditions of 300 K temperature and 1 atm pressure. In summary, it may be inferred that the interaction between proteins and hit chemicals results in an augmentation of the protein’s structural stiffness, hence enhancing its overall stability.

#### 3.2.4. Solvent-Accessible Surface Area (SASA)

**Figure 7:**
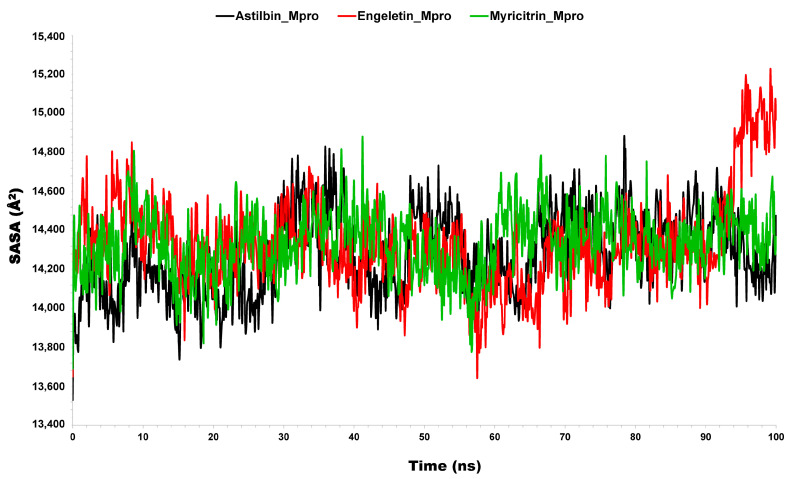
Representation of the SASA study for the selected complex structure of astilbin–Mpro, engeletin–Mpro (red), and myricitrin–Mpro (green).

Solvent-accessible surface area (SASA) is a measure of the surface area of a biomolecule (like a protein) or other molecular structures that solvent molecules can reach. It shows how enzyme–substrate complexes interact with solvents. It is defined as the extent to which atoms on the surface of a protein can form contact with the solvent, and it is generally measured in squared nanometers (nm^2^) [72,73]. It calculates the change in the conformation of the biomolecules during the period of the interactions [74,75]. Calculating the SASA of the protein–ligand complex predicts the total number of conformational changes that an aqueous solvent may access. Therefore, we determined the SASA to examine the complex–solvent interaction throughout the 100 ns MD simulation. From Figure 7, it can be seen that the average values of the SASA for the complex of Mpro with different ligands were 14,283.55, 14,329.47, and 14,335.72 Å (Table 1). These evaluations indicate that both of the complexes (engeletin–Mpro and myricitrin–Mpro) have a significantly similar value as compared to astilbin–Mpro with the value of 1483.55 Å. These findings give a satisfactory explanation for how the selected phytocompounds interact with the binding cavity of Mpro and how electrostatic forces help to form a stable binding interaction.

#### 3.2.5. Binding Free Energy (MM-PBSA) Calculation

**Figure 8:**
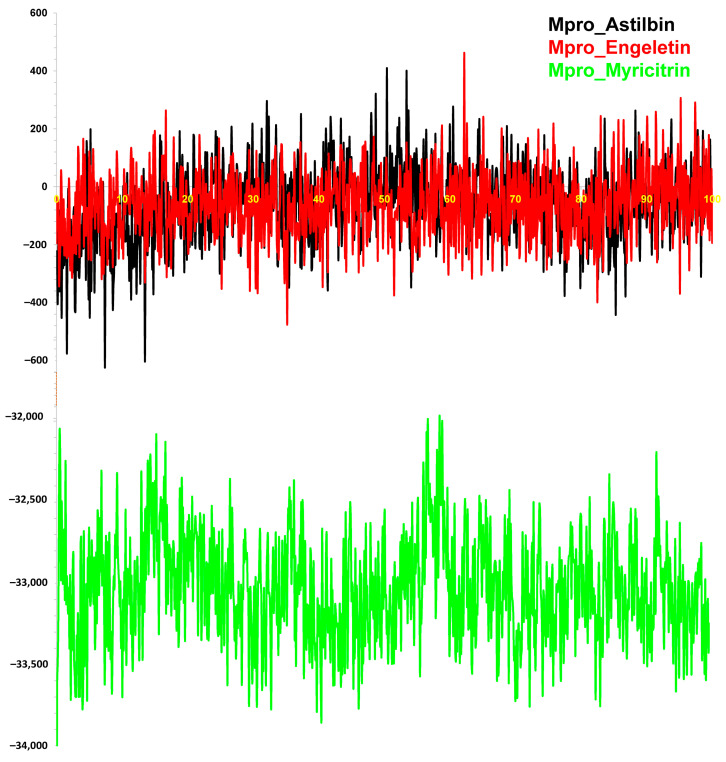
MM-PBSA binding energy calculation of myricitrin (green), engeletin (red) and astilbin (black) bound with Mpro calculated from the MD simulation trajectory.

To confirm the binding affinity of the molecular docking energy of the ligand–protein complex, we performed BFE analysis using MM-PBSA. This energy calculation of the complexes was analyzed from the 100 ns MD simulation trajectories through MM-PBSA methods. A total of 500 snapshot binding energies were calculated as a result, and the outcomes are plotted in Figure 8. The average BFE of the complexes, astilbin–Mpro, engeletin–Mpro, and myricitrin–Mpro, was −63.08, −60.88, and −33,042.16 kJ/mol, respectively (Table 1). The BFE of the docking complex was calculated to validate the affinity of the inhibitor for the receptor protein complex that was obtained from the docking study. The higher negative value of MMPBSA binding energy means better binding to the protein binding pocket [41,76]. Therefore, the experimental findings showed that the complexes astilbin–Mpro (−63.08 kJ/mol), engeletin–Mpro (−60.88 kJ/mol), and myricitrin–Mpro (−33,042.16 kJ/mol) had negative MMPBSA binding energies, which means that these complexes formed stable binding with the main protease SARS-CoV-2. Hence, these compounds might be used as potential inhibitors of SARS-CoV-2 infection. 

#### 3.2.6. Dynamic Cross-Correlation Matrix

**Figure 9:**
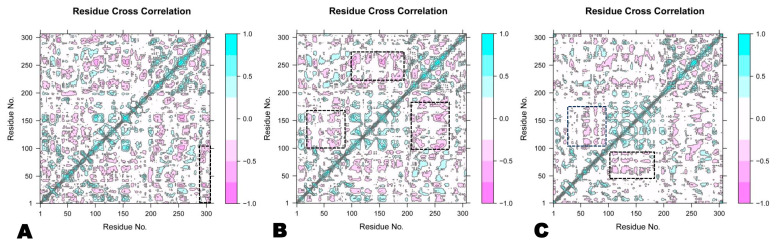
Ca-residue cross-correlation profiles for the myricitrin complex (**A**), engeletin complex (**B**), and astilbin complex (**C**).

The dynamic cross-correlation matrix (DCCM) was employed to analyze the relative motions of several simulation systems [77]. To explore the conformational change of the astilbin–Mpro complex, engeletin–Mpro complex, and myricitrin–Mpro complex, they were analyzed during a DCCM study. Figure 9 illustrates the fluctuations in the DCCM by displaying time-correlated information among the residues of the protein. The DCCM showed a strong connection ranging from −1.0 to 1.0, where −1.0 represents a dark purple color and 1.0 represents a dark blue color. Different shades of color represent different levels of correlation between residues, with darker colors indicating stronger association. The correlation coefficient, which ranges from −1 to 1, shows whether residues have a positive or negative link in their motions. A positive correlation means that the residues migrated together, while a negative correlation means that the residues moved in opposite directions [78]. Thus, the region of 0.5 to 1.0 represents a strong correlation, whereas the region of −0.5 to −1.0 shows a strong reverse correlation [79]. After examining the DCCM diagrams of the three systems, it could be observed that the correlated movements indicated by each complex were significantly different (Figure 9). This figure depicts that the collective movements of the astilbin complex have a positive correlation in the range of 1–100 residues, similar to the myricitrin complex, whereas the engeletin complex shows a positive correlation in the range of 100–175 residues like the other two systems. Thus, the complex of astilbin remains stable compared to the other two complexes of engeletin and myricitrin. Correlated movements within the complexes are especially apparent in the marked regions denoted by a dashed black outline.

#### 3.2.7. Principal Component Analysis (PCA)

**Figure 10:**
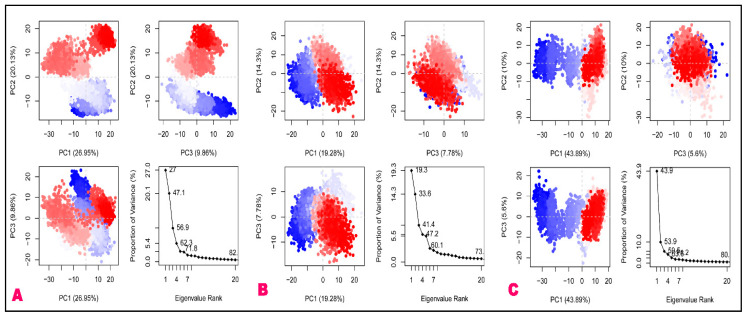
Graphical representation of the PCA analysis of the top-ranked complexes of (**A**) astilbin–Mpro, (**B**) engeletin–Mpro, and (**C**) myricitrin–Mpro, where, red and blue dots show the simulation’s illustration of protein conformational changes.

Principal component analysis (PCA) was used to make it easier to find and understand how important coordinated motions happen within different protein domains. A statistical method known as PCA is used to identify and extract the most significant dynamic movements in simulations. These motions are very important for biological processes to work properly. PCA can also be used to look into how different factors affect collective motion and to simplify motion, which is linked to the stability of the system and the functions of proteins. A variety of protein configurations are shown in the resulting graphs, with each dot representing a unique configuration. The arrangement of red and blue dots shows the simulation’s illustration of conformational changes. The progression from blue to white to red in the color spectrum represents the time it takes for the simulation to complete. The first timestep is represented by blue in Figure 10, whereas the middle timestep is represented by white. Lastly, the last timestep is represented by red.

Principal component analysis (PCA) revealed that the initial three principal components (PCs) account for a significant portion of the protein backbone motion observed in the molecular dynamics (MD) trajectories depicted in Figure 10. From this figure, it can be observed that the complexes of Mpro with astilbin, engeletin, and myricitrin exhibit distinct characteristics, as evidenced by the PCA. Each system of the Mpro complex contributed 27%, 19.3%, and 43.9% of the entire variation individually. The PC1 value of the myricitrin complex was determined to be the highest at 43.9%, indicating that the complex had undergone a greater number of conformational modifications, whereas the complex of engeletin exhibited the low value of 193%, indicating that it had been subjected to lower number of structural alterations. Additionally, it was observed that the PC1 of the astilbin structure exhibited a value of 27%, surpassing that of the engeletin complex. This finding suggests that the binding of astilbin plays a crucial role in stabilizing the structural alterations of the astilbin–Mpro complex.

#### 3.2.8. Gibbs Free Energy Landscape (FEL) Analysis

**Figure 11:**
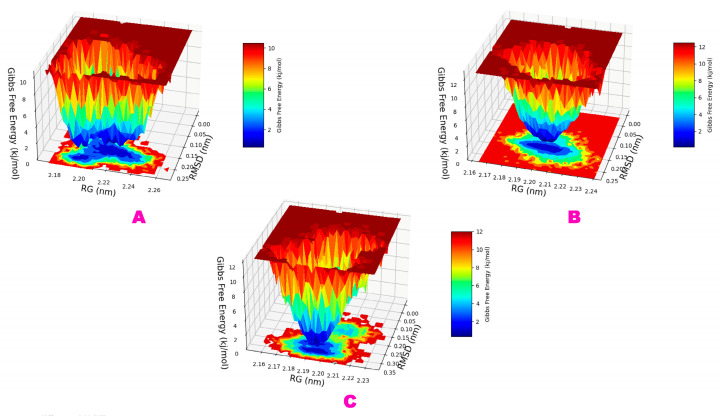
Graphical representation of Gibbs free energy landscape or the FEL of the (**A**) astilbin–Mpro, (**B**) engeletin–Mpro, and (**C**) myricitrin–Mpro complexes obtained from the dynamic simulation study.

Furthermore, FEL analysis was used to understand the conformational change of the overall systems. Figure 11 shows the free energy landscape (FEL) of finding the lowest free energy of the Cα backbone atoms of proteins in terms of RMSD and Rg. The complex of astilbin–Mpro achieved its lowest free energy (LFE) at 0.17 Å RMSD and 2.21 nm Rg, which is almost similar to the engeletin–Mpro complexes (Figure 11A,B). Because of its high stability and perfect conformation in the bound state of astilbin complex LFE, to gain insight into the receptor’s deterministic behavior toward the lowest energy state, the FEL was generated [80], whereas in the case of the myricitrin–Mpro complex, the ligand bound to the receptor Mpro to obtain the LFE and achieved the LFE at 0.27 Å RMSD and 2.19 nm Rg. Therefore, the FEL shows that the protein folds to reach its lowest energy state, which is properly reached because the ligands are bound. Additionally, the FEL of above complexes showed a deep basin over areas with higher free energy. The deep blue color regions (Figure 11) showed the local energy minima and actively promoted the stable conformations [81].

### 3.3. Pharmacokinetics Properties Analysis

#### 3.3.1. Drug-Likeness Profile (ADMET)

After the molecular docking study, the three top-ranked phytochemicals were investigated for drug-likeness prediction; the phytochemicals showed better pharmacokinetics properties and Lipinski’s rule of violation as compared to the FDA-approved drugs. As per Lipinski’s rule of five (RO5), these phytochemicals follow almost all of the parameters except for the number of hydrogen bond acceptors (HBA) ≤ 10 and the number of hydrogen bond donors (HBD) ≤ 5 (Table 2), and they successfully passed in the evaluation of Lipinski’s RO5. The compounds that showed better pharmacokinetics and followed all the rules of RO5 or at least two of the RO5 were considered drug-like compounds. Based on the RO5 violation and binding affinity score of the top-ranked phytochemicals, the drug-likeness properties of the screened phytochemicals are given in Table 2. The compound myricitrin followed only three of the RO5 and astilbin followed four out of five of the RO5, but the compound engeletin did not violate any of Lipinski’s rules. Therefore, our predicted molecules satisfied most of the drug-likeness properties. All of the candidate drug molecules were found to be lipophilic based on the predicted LogP value, with a consensus LogP value. The standard value of LogP for a drug-like molecules should be ≤5 [53], whereas the estimated values of LogP are within the range of 0.038, 0.194, and 0.333 for astilbin, myricitrin, and engeletin, respectively. The skin permeability or LogKp should be between −11.436 (cm/s) and −1.778 (cm/s) for standard drug-like phytocompounds [82], which means that the more negative the value of log Kp, the less permanent the molecule is in the skin. From Table 2, it can be observed that all of the values of LogKp for the top-ranked drugs are the same (−2.735 cm/s), which indicates that this could be considered as the acceptable range of skin permeability for therapeutic use [83]. The study also shows that the synthetic accessibility value for all of the chemicals is less than 10, suggesting that these molecules can be produced easily.

In this study, ADMET properties were studied for the three top-ranked phytocompounds in order to estimate their pharmacokinetic properties (Table 3). From Table 3, we can observe the pharmacokinetic properties (ADME/toxicity) of the selected phytocompounds. The findings reveal that these small chemicals have a high intestinal absorption (HIA) rate of more than 40% [84], which indicates that these phytochemicals show good absorption into the human intestine [55]. Caco-2 cell permeability is a significant parameter for an eligible candidate drug molecule. If any compound has a value of Papp > 8 × 10^−6^ cm/s, then it is considered as a high Caco-2 permeable compound. In the case of the pkCSM predictive dataset, high Caco-2 permeability would translate into predicted values > 0.90. A compound is considered to have a proper Cao-2 permeability if it has a predicted value > −5.15 log cm/s [55,85]. It was observed from the experimental data given in the table below Table 3 that all three compounds (astilbin, myricitrin, and engeletin) are likely to be well absorbed in the human gastrointestinal tract.

From the distribution catalog, the term volume of distribution (VDss) is defined as low if the value is lower than 0.71 L/kg and considered high when the value is higher than 2.81 L/kg [55]; on the other hand, the range for the value of logVDss is 0.04 to 20 L/kg [85]. From Table 3, it is clear that the selected compounds have excellent VDss values, which signifies that the selected compounds are distributed in the plasma very well. In the case of the blood–brain barrier (BBB), the standard value of Log BBB to cross the blood–brain barrier is 0 (excellent) to 1.0 (poor), while a value of less than zero implies that the selected compounds are not able to cross the BBB well. Moving to the CNS, or central nervous system, values, the compounds that have the value of logPs > −2 are able to penetrate the CNS whereas the compounds with a value logPs < −3 indicate that they are unable to penetrate the CNS. Therefore, it may be assumed that central nervous system penetration may have certain unfavorable effects. Regarding metabolism, almost 90% of commonly used drugs are digested by four isoenzymes, according to research. These are the CYP 1A2, CYP 2C9, CYP 2D6, and CYP 3A4 isoenzymes, with just a few interactions including the CYP 2B6, 2C8, and 2C19 isoenzymes [86]. According to Table 3, the selected phytocompounds may act as both CYP3A4 inhibitors and substrates. This enzyme is responsible for the majority of medicinal metabolism in the human organism system [87].

**Table 3:** Pharmacokinetic properties and toxicity analysis of the selected drug molecules computed using the pkCSM web-tool [55,87].

Compounds	Absorption		Desorption			Excretion	Metabolism CYP							Toxicity	
	Caco2 Log cm/s	HIA	BBB	VDss	CNS	TC	Substrate		Inhibitors					AMES	Skin Sensitization
							2D6	3A4	1A2	2C19	2C9	2D6	3A4		
Astilbin	0.34	49.00	−1.19	1.59	−4.17	−0.28	No	No	No	No	No	No	No	No	No
Myricitrin	−0.98	43.33	−1.81	1.55	−4.37	0.30	No	No	No	No	No	No	No	No	No
Engeletin	0.41	58.66	−0.99	1.12	−4.01	0.05	No	No	No	No	No	No	No	No	No

According to the excretion profile, these phytocompounds can be excreted from the bloodstream in a relatively effective manner with virtually no risk of drug formation in the organism because the results of the study demonstrated that these phytochemicals had a total clearance (TC) value of 0.5 (log mL min^−1^ kg^−1^), with the only exception being astilbin, which had a negative value of −0.28. From the toxicity analysis, the AMES toxicity parameter was estimated and showed no mutagenicity of the selected phytocompounds. The top-ranked drug molecules also showed no skin sensitization to human skin, which is a significant safety concern. In conclusion, these substances have favorable in silico pharmacokinetic characteristics and will not injure people or make them ill; this, their therapeutic effectiveness against SARS-CoV-2 is confirmed.

#### 3.3.2. Biological Activity Analysis

The possible biological activities of the top-ranked phytocompounds were investigated, such as G protein-coupled receptor (GPCR) ligand activity, protease inhibition, ion channel inhibition, kinase inhibition, nuclear receptor ligand activation, and enzyme inhibition. A cheminformatics-based web tool, “Molinspiration”, was used to calculate biological activity, where values greater than 0 indicate significant biological activity, values between −0.5 and 0 indicate moderate activity, and values less than −0.5 indicate inactivity [88]. It was observed that astilbin and engeletin had a larger GPCR value for ligand activity than myricitrin (Table 4), which indicates that astilbin and engeletin have better ligand activity or are potent inhibitors [89,90], but in some cases, a negative value may indicate that the compound has an inhibitory effect, but it is difficult to say for sure without further confirmation. Again, astilbin and engeletin showed better ion channel inhibitory activity whereas myricitrin showed less ion channel inhibition but comparatively greater kinase inhibition and nuclear receptor ligand activity compared to the other two phytocompounds. Myricitrin also shows higher enzyme inhibitory activity than astilbin and engeletin, but the protease inhibition activity of myricitrin is lower than these two compounds. Thus, there are no compounds that cross the standard range less than −0.5, and all of our suggested compounds are biologically active against all targets.

### 3.4. Density Functional Theory (DFT)

#### 3.4.1. Frontier Molecular Orbital Study

**Figure 12:**
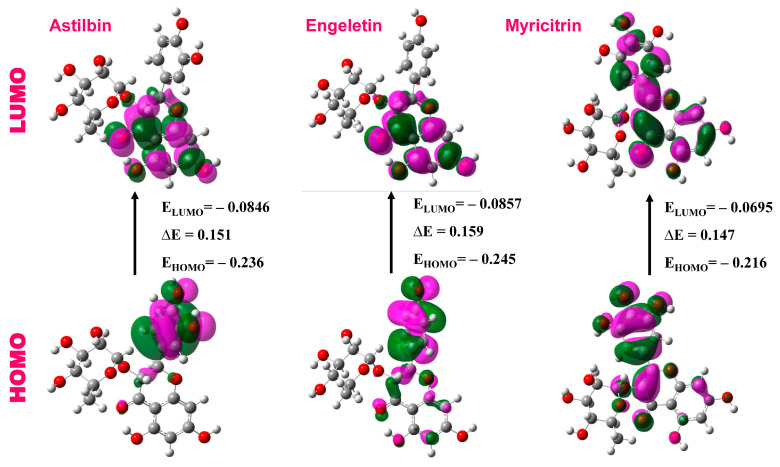
The HOMO and LUMO molecular orbitals of the selected candidate drug molecules.

The frontier molecular orbital analysis was performed to predict the reactivity and the reactive region in a compound′s molecular system. The HOMO and LUMO molecular orbitals are depicted in Figure 12. A standard measure for evaluating a compound’s chemical reactivity is the energy gap between its highest occupied molecular orbital (HOMO) and lowest unoccupied molecular orbital (LUMO). A molecule is chemically unstable and shows low reactivity if there is a significant energy difference between its LUMO and HOMO levels. The main cause of this phenomenon is the electronic transition being slowed down by a substantial energy gap between the lowest and highest energy states. These parameters are essential to understanding the magnitude of ligand interaction with the receptor-binding pocket [61,91].

In general, a lower HOMO–LUMO energy gap indicates that the compound is stable [56,58,89]. Table 5 shows that the HOMO energy levels range from −0.230 eV to −0.246 eV, and the LUMO energy levels range from −0.0436 eV to 0.092 eV. Myricitrin has a lower energy gap (0.147), indicating that it is more reactive than astilbin and engeletin, which have higher ∆E values.

#### 3.4.2. Quantum Chemical Descriptors

Any organic compound or physiologically active molecule has a specific relevance for chemical descriptors. Koopmans’ theorem represents a theoretical approach for connecting the chemical activity of structures of molecules to their electric characteristics [92]. This theorem could be used to determine the reactivity of a molecule using the quantum chemical descriptors, which include the HOMO and LUMO energy gap (∆E), ionization potential (I), affinity for electrons (A), chemical potential (µ), electronegativity (χ), hardness (η), electrophilicity (ω), and softness (S). These numbers were calculated using the DFT tool. 

Table 5 represents the HOMO and LUMO energies, electrophilic index, chemical hardness, softness, chemical potentiality, electron affinity, and energy gap (∆E) of the top-ranked phytocompounds. These numerical profiles were evaluated by using density functional theory (DFT) in Gaussian 09. The energy generated when a molecule in its ground state accepts an electron is known as electronic affinity (A). When it comes to pharmacological compounds, electron affinity levels may provide data on the molecule’s activity and chemical reactivity. 

A molecule’s ability to take electrons is indicated by a higher electron affinity value, which could have an impact on how it interacts with other molecules or biological systems. The compound engeletin has a higher A value with (0.0857 eV) than astilbin (0.0846 eV) and myricitrin (0.0695 eV). As before, “softness” (σ) and “hardness” (η) serve as important determinants of a molecule’s behavior in a chemical process. Compared with structures found in previous research, the obtained results indicated a high hardness value and a low softness value [93]. The ability of a molecule to attract electrons is measured by its electronegativity (χ) [93]. All compounds were shown to be stronger electrophiles than the conventional medicines, as demonstrated by their higher electrophilicity index ω values [94,95]. Thus, Table 4 shows that all of the compounds at the top had strong electrophilic indexes in addition to other physicochemical characteristics.

## 4. Conclusions

In spite of the fact that there are several promising therapies for the treatment of SARS-CoV-2 infections, most of them are synthetic drugs that may have some adverse effect on the human body. Therefore, it is necessary to explore potential natural medicines instead of synthetic drugs, since natural medicines have fewer adverse effects compared to synthetic drugs. Although *P. alliacea* L. is a well-known medicinal plant that contains potential phytochemicals that are considered natural medicine for various diseases, so far, there is no study in the literature that has explored the medicinal properties of *P. alliacea* L. for use against SARS-CoV-2 infections. Therefore, this study investigated the anti-SARS-CoV-2 activity of *P. alliacea* L.-mediated phytocompounds through bioinformatics analysis. In order to explore potential phytocompounds from *P. alliacea* L. as candidate drug molecules through molecular docking analysis, at first, we considered the infection-causing main protease (Mpro/3CLpro) of SARS-CoV-2 as the receptor protein in this study. Then, the three top-ranked Mpro-guided phytochemicals (myricitrin, engeletin, and astilbin) of *P. alliacea* L. were selected for the treatment of SARS-CoV-2 infections based on their binding affinity scores and non-bonding interaction against the target protein (Mpro). The docking results (binding scores < −7.0 Kcal/mol) also showed that at least one of these three phytocompounds is able to inhibit the other top-ranked SARS-CoV-2 infection-related key-genes/proteins (ACE2, spike, NFKBIA, MAPK8, N, TNF, TMRSS2, RdRp, PLpro, and IL6) that were selected during the systematic literature review. Then the 100 ns MD simulation results with 1.81 Å < RMSD < 2.40 Å, 1.30 Å < RMSF < 1.40 Å, 14,283.55 Å < SASA < 14,335.72 Å, −1.0 < DCCM > 1.0, 19.3% < PCA < 43.9%, and −60.88 < MM-PBSA binding free energy < −33,042.16 kJ/mol and 0.17 Å < Gibbs free energy landscape (FEL) < 0.27 Å indicated the structural stability and flexibility of the three top-ranked protein–ligand complexes. The ADME/T and DFT analysis results indicated that the proposed phytocompounds possess good pharmacokinetic properties. All of the phytocompounds follow the LRO5, especially the LogP, which falls within the range (<5) of 0.038, 0.194, and 0.333 for astilbin, myricitrin, and engeletin, respectively, and all of the compounds were found to have non-toxicity. However, to discover effective therapeutic candidates from *P. alliacea* L. against SARS-CoV-2 infections, it is necessary to validate our predicted top-ranked phytochemicals by conducting in-vitro, in-vivo, pre-clinical, and clinical trials to determine their practical utility.

## Figures and Tables

**Table 1:** The average values of RMSDb, RMSF, radius of gyration (Rg), SASA, number of H-bonds, and binding free energy for all protein–ligand complexes. The values within the first bracket (.) indicate the standard deviation (SD).

Name of Complex	MD Simulation Study					
	Average RMSD (SD)	Average RMSF (SD)	Average Rg (SD)	Average SASA (SD)	Average H-Bonds (SD)	Average Binding Energy (SD)
Mpro vs. Astilbin	1.81 (0.30)	1.40 (1.04)	22.34 (0.16)	14,283.55 (213.76)	12.82 (1.91)	−63.08 (133.93)
Mpro vs. Engeletin	2.40 (0.35)	1.32 (0.93)	22.24 (0.16)	14,329.47 (234.89)	12.19 (1.71)	−60.88 (117.51)
Mpro vs. Myricitrin	2.16 (0.25)	1.30 (0.82)	22.32 (0.12)	14,335.72 (163.21)	12.34 (1.93)	−33,042.16 (556.96)

**Table 2:** Drug-likeness properties of the three phytochemicals.

Compound	Molecular Weight	LogP_o/w_	NHBA	NHBD	Log Kp (Cm/S)	Lipinski’s Rule		Synthetic Accessibility
						Follow	Violation	
Astilbin	450.396	0.038	10	7	−2.735	4	1	5.27
Myricitrin	464.379	0.194	12	8	−2.735	3	2	5.32
Engeletin	434.397	0.333	10	5	−2.735	5	0	5.20

**Table 4:** Biological activity data of the top-ranked drug molecules.

Compounds	GPCR Ligand	Ion Channel Inhibitor	Kinase Inhibitor	Nuclear Receptor Ligand	Protease Inhibitor	Enzyme Inhibitor
Astilbin	0.11	0.05	0.03	0.12	0.15	0.33
Myricitrin	−0.02	−0.08	0.08	0.14	−0.06	0.38
Engeletin	0.10	0.03	0.04	0.11	0.17	0.34

**Table 5:** Calculated FMO energies and their corresponding physicochemical descriptors (chemical hardness, electronegativity, softness, chemical potential, and global electrophilicity index).

Parameter	Astilbin	Engeletin	Myricitrin
Optimized energy (a.u.)	−1640.846	−1565.628	−1714.364
Dipole moment, D	3.1739	2.8213	5.0897
HOMO energy (E_HOMO_)	−0.236	−0.245	−0.216
LUMO energy (E_LUMO_)	−0.0846	−0.0857	−0.0695
Energy gap (∆E = E_LUMO_ − E_HOMO_)	0.151	0.159	0.147
Ionization potential (I)	0.236	0.245	0.216
Electron affinity (A)	0.0846	0.0857	0.0695
Chemical hardness (η)	0.0757	0.0797	0.0733
Softness (σ)	13.21	12.55	13.64
Electro-negativity (χ)	0.160	0.165	0.142
Chemical potential (μ)	−0.160	−0.165	−0.142

## Data Availability

Data are contained within the article and Supplementary Material.

## Supplementary Materials

The following supporting information can be downloaded at: https://www.mdpi.com/article/10.3390/molecules29112524/s1. Table S1: Highest Binding affinity Score of top 35 phytocompounds of *P. alliacea* against Mpro. Table S2: Molecular docking result of the three top-ranked phytochemicals with another independent target receptor of SARS-CoV-2. Table S3: Tabular representation of conventional hydrogen bonding and its bond length for top-ranked protein–ligand complexes. Table S4: Selection of top-ranked SARS-CoV-2 infection-causing ten key proteins/proteases identified by the literature review of 57 articles [1,2,3,4,5,6,44,96,97,98,99,100,101,102,103,104,105,106,107,108,109,110,111,112,113,114,115,116,117,118,119,120,121,122,123,124,125,126,127,128,129,130,131,132,133,134,135,136,137,138,139,140,141,142,143,144,145].
