# Supplementary material for: Exploring the Therapeutic Potential of Petiveria alliacea L. Phytochemicals: A Computational Study on Inhibiting SARS-CoV-2’s Main Protease (Mpro)
Source: Molecules. 2024 May 27;29(11):2524. doi: 10.3390/molecules29112524 (PMC11173994; doi:10.3390/molecules29112524)
Supplement: Supplementary file 1 [file molecules-29-02524-s001.zip › molecules-2986372-supplementary.pdf]

Exploring the Therapeutic Potential of Petiveria alliacea L. Phytochemicals: A Computational Study on Inhibiting SARS-CoV-2's Main Protease (Mpro)

Md. Ahad Ali [1,2], Humaira Sheikh [3], Muhammad Yaseen [4,*], Md Omar Faruqe [5], Ihsan Ullah [4], Neeraj Kumar [6], Mashooq Ahmad Bhat [7] and Md. Nurul Haque Mollah [1,*]

[1]Bioinformatics Laboratory, Department of Statistics, Faculty of Science, University of Rajshahi, Rajshahi 6205, Bangladesh; ahad.chembd@gmail.com
[2]Department of Chemistry, Faculty of Science, University of Rajshahi, Rajshahi 6205, Bangladesh
[3]Department of Chemistry, Faculty of Science, Bangabandhu Sheikh Mujibur Rahman Science & Technology University, Gopalganj 8100, Bangladesh; humairasheikh.bd@gmail.com
[4]Institute of Chemical Sciences, University of Swat, Main Campus, Charbagh 19130, Pakistan; ihsanmtk@uswat.edu.pk
[5]Department of Computer Science and Engineering, Faculty of Engineering, University of Rajshahi, Rajshahi 6205, Bangladesh; faruqe.cse@gmail.com
[6]Department of Pharmaceutical Chemistry, Bhupal Nobles' College of Pharmacy, Udaipur 313001, Rajasthan, India; neerajkumarkamra@gmail.com
[7]Department of Pharmaceutical Chemistry, College of Pharmacy, King Saud University, Riyadh 11451, Saudi Arabia; mabhat@ksu.edu.sa
*Correspondence: muhammadyaseen.my907@gmail.com (M.Y.); mollah.stat.bio@ru.ac.bd (M.N.H.M.)

Table of contents for supplementary data

S. N.	Contents	Page No.
01	**Table S1**: Highest Binding affinity Score of top 35 phytocompounds of *P. alliacea* against Mpro.	2 – 3
02	**Table S2**: Molecular docking result of top ranked three phytochemicals with another independent target receptor of SARS CoV-2.	3
03	**Table S3**: Tabular representation of conventional hydrogen bonding and its bond length for top-ranked protein–ligand complexes	3
04	**Table S4:** Selection of top-ranked SARS-CoV-2 infection-causing ten key proteins/proteases identified by the literature review of 57 articles [1-6,44,96-145].	4 – 6

Supplementary Files:

Table S1: Highest Binding affinity Score of top 35 phytocompounds of *P. alliacea* against Mpro.

Plants Name	Name	PubChem ID	Molecular Formula	Molecular Weight	Binding Affinity
petiveria alliacea L.	Myricitrin	5281673			-8.9
	Friedelanol	101341			-8.9
	Rutin	5280805			-8.7
	Engeletin	6453452			-8.7
	Astilbin	119258			-8.5
	Isoquercitrin	5280804			-8.5
	Ursolic Acid	64945			-8.1
	Stigmasterol	5280794			-7.9
	Isoaborinol	12305182			-7.9
	Apigenin	5280443			-7.7
	Kaempferol	5280863			-7.7
	Naringenin	932			-7.6
	Barbinervic Acid	194183			-7.6
	Beta-sitosterol	222284			-7.5
	Luteolin	5280445			-7.4
	isoarborinol-acetate	21582933			-7.4
	Lupeol	259846			-7.3
	Daucosterol	5742590			-7.3
	Quercetin	5280343			-7.2
	Dihydroquercetin	439533			-7.1
	Leridol	10495449			-7
	spathulenol	92231			-6.5
	Coumarin	323			-5.7
	trans-stilbene	638088			-5.7
	Allantoin	204			-5.3

	carvacrol	10364			-5.2
	cinnamaldehyde	637511			-5.2
	dibenzyl sulphide	10867			-5.1
	Beta-Pinene	14896			-5
	Alpha-Pinene	6654			-4.9
	Nerolidol	5284507			-4.9
	Benzyl disulfide	9012			-4.7
	Benzyl trisulfide	122842			-4.7
	benzaldehyde	240			-4.6
	Phytol	5280435			-4.6

Table S2: Molecular docking result of top-ranked three phytochemicals with other independent target receptor of SARS CoV-2.

COMPOUND	Binding affinity /(Kcal/mol)									
	S	MAPK8	RdRp	ACE	TMPRSS2	N	PLpro	IL6	TNF	NFKBIA
Myricitrin	-8.5	-9.0	-8.5	-8.0	-7.7	-8	-7.4	-6.6	-7.1	-6.3
Astilbin	-8.7	-7.9	-8.4	-7.8	-7.6	-8	-6.7	-6.6	-6.4	-6.3
Engeletin	-7.8	-7.8	-7.6	-7.8	-7.5	-6.7	-7.4	-6.9	-6.4	-6.2

Table S3: Tabular representation of conventional hydrogen bonding and its bond length for top-ranked protein–ligand complexes

**Name**	**SARS-CoV-2 Protein 1(3CL^{pro})**	
	**Vital residues for H-bond formation**	**Length (in Å)**
Myricitrin	ASN^{151}	1.99
	THR^{111}	3.00
	GLN^{110}	3.12
Friedelanol	THR^{111}	3.08, 2.94
Engeletin	THR^{111}	3.20, 2.97
	ASN^{151}	2.14
Astilbin	ASN^{151}	2.46

Table S4: Selection of top-ranked SARS-CoV-2 infection-causing ten key proteins/proteases identified by the literature review of 57 articles [1-6,44,96-145].

**Article references**	List of key proteins/proteases
Verma et al., 2021 [1]	**PLpro, Mpro/3CLpro**
Astuti et al., 2020 [2]	**ACE2, Mpro, PLpro, Spike, TMPRSS2, RdRp, IL6**, nsp1-16, and etc.
Wang et al., 2022 [3]	**3CLpro**
Sharma et al., 2022 [4]	**Mpro**
Narayanan et al., 2022 [5]	**Mpro, PLpro**
Hassam et al., 2022 [6]	**Mpro**
Ahmad et al., [44]	**ACE2**
Kuo et al., 2021 [96]	**3CLpro, PLpro**
Cavasotto and Di Filippo, 2021 [97]	**Mpro, PLpro, S**
Gil et al., 2020 [98]	**NFKB1A, 3CLpro, PLpro, S**
Guedes et al., 2021 [99]	**PLpro, Mpro, RdRp, N, S**
Liang et al., 2021 [100]	**RdRp, 3CLpro, PLpro, S**
Rahman et al., 2021 [101]	**Mpro, RdRp, PLpro, S**
Murugan et al., 2020 [102]	**3CLpro, PLpro, RdRp**
Manikyam and Joshi, 2020 [103]	**3CLpro, PLpro, RdRp**
Wu et al., 2020 [104]	**3CLpro, PLpro, RdRp**
Abdel-Basset et al., 2020 [105]	**3CLpro, RdRp**
Wang et al., 2021 [106]	NFKB1, CHUK, MAPK3, MAPK1, **NFKB1A**, CASP3, **IL6**, MAPK8, BAX, and **TNF**, **TMPRSS2**, **ACE2, 3CLpro, RdRp**, **PLpro, Spike**
Mishra et al., 2021) [107]	**S, ACE2, 3CLpro**, CTSL, nucleocapsid protein, **RdRp**, NSP6
Nelakuditi and Shrivastava, 2020 [108]	**Mpro, S, ACE2, RdRp**
Mhatre et al., 2021 [109]	**3CLpro, S, PLpro, RdRp, ACE2**
Joshi et al., 2020 [110]	**Mpro, RdRp and ACE2**
Shi et al., 2021 [111]	**ACE2, Mpro, RdRp**
Panda et al., 2020 [112]	**Mpro, S,** RBD, **ACE2**

Jena et al., 2021 [113]	**ACE2, Mpro**
Tao et al., 2020 [114]	**ACE2, 3CLpro**
Duverger et al., 2021 [115]	**ACE-2**
Xiang et al., 2021 [116]	**ACE2**
Li and Yang, 2020 [117]	BALF, **ACE2**
Han et al., 2020 [118]	**IL6, ACE2**
de Oliveira et al., 2021 [119]	**ACE2**
Bardaweel et al., 2021[120]	DPP4, **ACE2**
Kabir et al., 2021 [121]	**ACE2**, **TMPRSS2**
Bojkova et al., 2020 [122]	**ACE2**, **RdRp**
Aftab et al., 2020 [123]	**RdRp**, ASP760, ASP761,
Elfiky, 2021 [124]	**RdRp**
Pirzada et al., 2021 [125]	**RdRp**
Agrawal et al., 2021 [126]	**S, RdRp**
Y. J. Sun et al., 2021 [127]	**TMPRSS2**
Cho et al., 2021 [128]	**TMPRSS2**
Gao et al., 2021 [129]	**PLpro**
Zhao et al., 2021 [130]	**PLpro**
Weglarz-Tomczak et al., 2021 [131]	**PLpro**
Yang et al., 2021 [132]	**S** protein
Jeon et al., 2020 [133]	**N** protein
F. Liu et al., 2021 [134]	AKT1, TP53, **TNF, IL6**, BCL2L, ATM
G. Li et al., 2021 [135]	**TNF**
Alanazi, Farah, and Hor 2022 [136]	NSP1, **PLpro**, **Mpro**, NSP9, **RdRp**, NSP13, NSP15, ORF3a, **S**, E, M, ORF6, ORF7a, **N**
Jose et al. 2022 [137]	**S**
Smarajit et al. 2020 [138]	**N**, NSP2, **Mpro**, IL1, **IL6**
Xu et al. 2021 [139]	**ACE2**
Ruchi et al. 2020)	**ACE2**

[140]	
Ahmad et al. 2022 [141]	**RdRp**
Maiti, Banerjee, and Kanwar 2020 [142]	ACE1, **ACE2**, AT1 and AT2 and **Spike**
Khan et al. 2022 [143]	**3Clpro** and NSP5
S. Ullah et al. 2022 [44]	**S & ACE2**
Ullah et al. 2022 [144]	**3Clpro**
Siddiqui et al. 2022 [145]	**S**, NRBD, **3Clpro**, **PLpro**
**Selection of top ranked 10 key proteins/proteases based on larger frequency across the lists of key proteins**	**ACE2, 3CLpro, S, PLpro, RdRp, IL6, TNF, TMPRSS2, NFKB1A, N**
